# A novel capsid protein network allows the characteristic internal membrane structure of *Marseilleviridae* giant viruses

**DOI:** 10.1038/s41598-022-24651-2

**Published:** 2022-12-11

**Authors:** Akane Chihara, Raymond N. Burton-Smith, Naoko Kajimura, Kaoru Mitsuoka, Kenta Okamoto, Chihong Song, Kazuyoshi Murata

**Affiliations:** 1 Department of Physiological Sciences, School of Life Science, The Graduate University for Advanced Studies (SOKENDAI), Okazaki, Aichi Japan; 2 Exploratory Research Center on Life and Living Systems (ExCELLS), National Institutes of Natural Sciences, Okazaki, Aichi Japan; 3 National Institute for Physiological Sciences, National Institutes of Natural Sciences, 38 Nishigonaka, Myodaiji, Okazaki, Aichi 444-8585 Japan; 4 Research Center for Ultra-High Voltage Electron Microscopy, Osaka University, Ibaraki, Osaka Japan; 5 Program in Molecular Biophysics, Department of Cell and Molecular Biology, Uppsala University, Uppsala, Sweden

**Keywords:** Biological techniques, Microbiology, Structural biology

## Abstract

*Marseilleviridae* is a family of giant viruses, showing a characteristic internal membrane with extrusions underneath the icosahedral vertices. However, such large objects, with a maximum diameter of 250 nm are technically difficult to examine at sub-nanometre resolution by cryo-electron microscopy. Here, we tested the utility of 1 MV high-voltage cryo-EM (cryo-HVEM) for single particle structural analysis (SPA) of giant viruses using tokyovirus, a species of *Marseilleviridae*, and revealed the capsid structure at 7.7 Å resolution. The capsid enclosing the viral DNA consisted primarily of four layers: (1) major capsid proteins (MCPs) and penton proteins, (2) minor capsid proteins (mCPs), (3) scaffold protein components (ScPCs), and (4) internal membrane. The mCPs showed a novel capsid lattice consisting of eight protein components. ScPCs connecting the icosahedral vertices supported the formation of the membrane extrusions, and possibly act like tape measure proteins reported in other giant viruses. The density on top of the MCP trimer was suggested to include glycoproteins. This is the first attempt at cryo-HVEM SPA. We found the primary limitations to be the lack of automated data acquisition and software support for collection and processing and thus achievable resolution. However, the results pave the way for using cryo-HVEM for structural analysis of larger biological specimens.

## Introduction

The “giant viruses” are exceptionally large physical size viruses, larger than small bacteria^1^. They also have a much larger genome (> 100 kilobases (kb)) than other viruses and contain many genes (> 50 genes) not found in other viruses^2^. One of the characteristics of these viruses is that they possess double-stranded DNA encapsulated in a lipid bilayer^3^. These large DNA viruses are now taxonomically classified into the phylum *Nucleocytoviricota*^4^, but have historically been referred to be nucleo-cytoplasmic large DNA virus (NCLDV). NCLDVs are an expansive clade of large viruses that possess double-stranded DNA and target varying host eukaryotes^5^. NCLDVs are composed of several families, including the *Asfarviridae*, *Ascoviridae*, *Iridoviridae*, *Marseilleviridae*, *Mimiviridae*, *Phycodnaviridae*, and *Poxviridae*, and unclassified viruses such as cedratviruses, faustoviruses, medusaviruses, *Mininucleoviridae*, molliviruses, orpheoviruses, pacmanviruses, pandoraviruses, and pithoviruses^6^. Even now, a variety of NCLDVs are isolated and studied from around the world. A new order, *Megavirales*, has been proposed based on the shared characteristics of these viruses^7^. NCLDVs exhibit several types of shapes depend on the species^2^. *Asfarviridae*, *Ascoviridae*, *Iridoviridae*, *Marseilleviridae*, *Mimiviridae*, *Phycodnaviridae*, faustoviruses, medusaviruses, pacmanviruses, and *Mininucleoviridae* exhibit an icosahedral shape. *Poxviridae* exhibits a brick shape. Cedratviruses, molliviruses, orpheoviruses, pandoraviruses, and pithoviruses exhibits an amphora shape. Sizes vary also; the amphora-shaped pithoviruses are the largest of the giant viruses, exceeding 2 μm in size, but there is a significant variation in the actual dimensions^8^. On the other hand, the icosahedral-shaped mimivirus is ~ 500 nm in diameter (not including fibrous filaments extending from the capsid)^9^, and its closest known relative cafeteriavirus is ~ 300 nm^10^. The brick-shaped poxviruses are approximately 350 × 270 nm, although precise dimensions are variable^11^. Other icosahedral viruses are ~ 260 nm for medusavirus^12,13^, ~ 180 nm for iridovirus^14^, ~ 190 nm for PBCV-1^15^. ASFV is ~ 250 nm, but is complicated because it has an external membrane^16^.

*Marseilleviridae* is a family of the new order of NCLDVs^17^, which have a highly complex ~ 360 kb genome and a particle size of ~ 250 nm. The first member, Marseillevirus, was isolated in 2007 by culturing a water sample from a cooling tower in Paris, France^18^. Currently, Cannes 8 virus, Melbournevirus, Marseillevirus, Tokyovirus, Port-Miou virus, Lausannevirus, Noumeavirus, Insectminevirus, Tunisvirus, Brazilian marseillevirus, Golden mussel marseillevirus, and more species belong to this family, and these are classified into five lineages, A to E^19,20^. Several studies have also reported the presence of *Marseilleviridae* in humans^21–24^. However, there is controversy surrounding marseillevirus infections of humans as other studies have shown no evidence^25,26^. Melbournevirus, one of the *Marseilleviridae* belonging to lineage A^27^, has previously been analysed by cryo-electron microscopy (cryo-EM) single particle analysis (SPA) at 26 Å resolution^28^. It shows the icosahedral capsid with a triangulation number of T = 309, shows the characteristic internal membrane inside the capsid that extrudes just underneath the vertices, and the unique large density body inside the nucleoid. Tokyovirus is the first *Marseilleviridae* isolated in Asia in 2016 and is classified in lineage A^29^. Tokyovirus was utilised to investigate the characteristic capsid structure of the icosahedral *Marseilleviridae* in this study.

Few studies have revealed the capsid structure of large icosahedral viruses in detail. An electron microscopy study half a century ago showed that the large icosahedral virus capsid is composed of 20 and 12 sets of trisymmetron and pentasymmetron, respectively^30,31^, although these viruses are not classified as NCLDVs. Each of these is a cluster of capsomers formed by pseudo-hexamer. In recent years, the structures of many viruses have been revealed with high resolution by using cryo-EM SPA for determination of overall structure^32^, dynamics^33,34^, and assembly^35^. However, the large icosahedral viruses present special challenges for high resolution cryo-EM simply from their size, which imposes hard limits on sample preparation, data acquisition and image reconstruction techniques. A hard limit is one which cannot be overcome using the same conditions, e.g.: particle count per micrograph, or the resolution limit imposed by the Nyquist frequency. A soft limit can be overcome, e.g., by collecting more micrographs, but a hard limit cannot. Therefore, several different methods including cryo-electron tomography, scanning electron microscopy, and atomic force microscopy, were initially combined with cryo-EM and investigated the structure of giant viruses^9,36^. The different methods which have been used to investigate giant viruses have been examined before^37^. However, in 2019, the structure of *Paramecium bursaria* chlorella virus 1 (PBCV-1) at 3.5 Å resolution^15^ and the structure of African swine fever virus (ASFV) at 4.1 Å resolution^16^ were reported by the use of cryo-EM SPA, respectively. These cryo-EM SPA of the large icosahedral viruses at high resolution have commonly used microscopes with an accelerating voltage of 300 kV. However, these reports utilise a method called “block-based reconstruction”^38^ to achieve these resolutions which focusses on sub-sections (“blocks”) of the virus to permit localised defocus refinement, resulting in reconstructions of higher resolution. In these reports, it was shown that the capsids of PBCV-1 and ASFV are composed of one “major” capsid protein (MCP) and a combined fourteen kinds (PBCV-1) or four kinds (ASFV) of “minor” capsid protein (mCP), respectively. The MCP of PBCV-1 and ASFV includes double “jelly roll” motifs, each of which consists of eight β strands^39^. The connecting loops in the motifs and their glycosylation sites provide these viruses with unique functions. The mCPs form a hexagonal network to fill the space between the pseudo-hexagonal MCP trimers, which contribute to keeping the capsid structure stable. Further, PBCV-1 and ASFV possess an mCP called a “tape-measure” protein, which is a long filamentous protein that extends from a pentasymmetron to an adjacent pentasymmetron along the trisymmetron edge. It was proposed that the length of this tape-measure protein determines the capsid size^40^.

**Figure 1:**
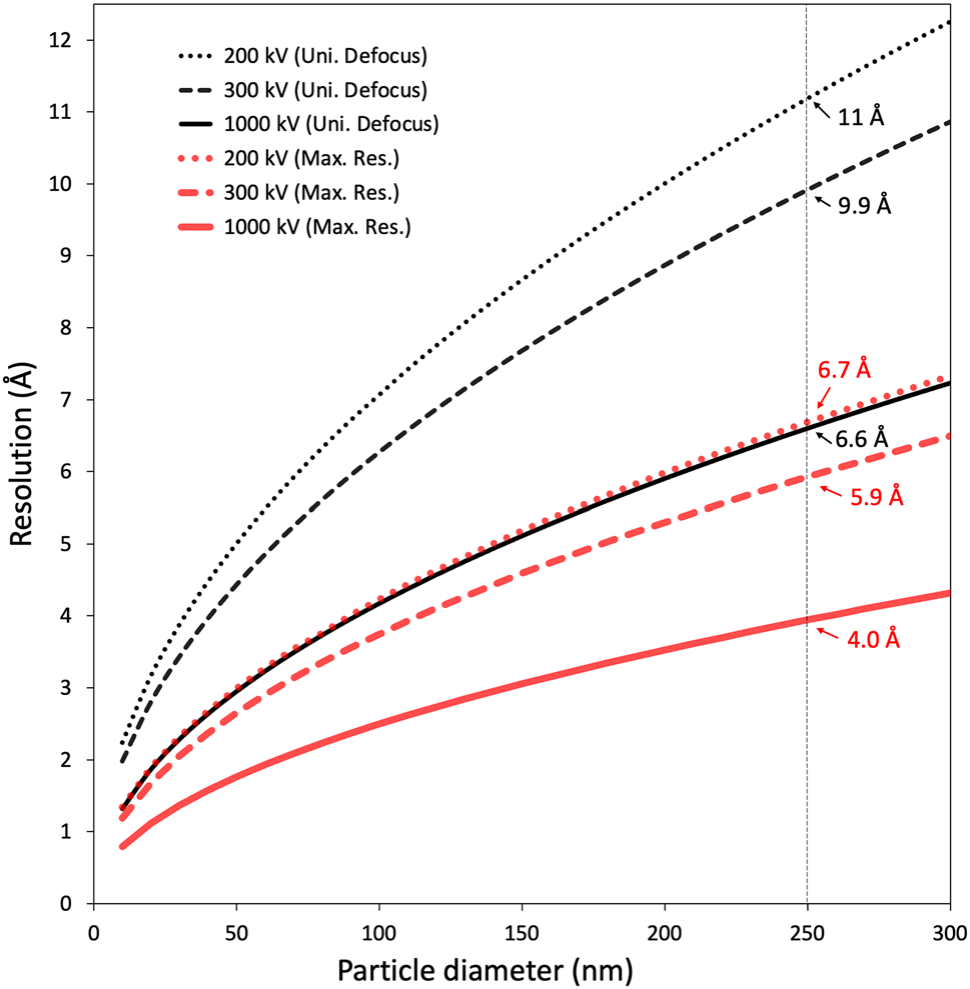
The depth of field effect in cryo-EM. Theoretical resolution limits caused by the size of particles (Eq. 1) are plotted at accelerating voltages of 200, 300 and 1000 kV, assuming uniform defocus (Black curves). The resolutions at which phase error limits the theoretical maximum resolution of a cryo-EM reconstruction (Eq. 2) are plotted at accelerating voltages of 200 kV, 300 kV and 1000 kV (Red curves). Dashed vertical line drawn at the maximum diameter (250 nm) of tokyovirus. Theoretical resolution limits at the maximum diameter of tokyovirus are shown to one decimal place.

Herein we focus on the single particle reconstruction of the entire tokyovirus using 1 MV cryo-HVEM (high-voltage electron microscopy). HVEM was originally developed to extend attainable resolution using shorter electron wavelengths^41^. Major usage is currently focussed on thick specimens, which lower acceleration voltages are unable to penetrate^42^. Cryo-HVEM on biological samples has not previously been reported for single particle analysis, and only a few examples using tomography have been reported^8,43,44^. For thick samples (e.g., tokyovirus with a maximum diameter of 250 nm), the influence of depth of field causes an internal focus shift, imposing a hard limit on attainable resolution^45^. However, increasing the accelerating voltage (shortening the wavelength of the emitted electrons) can increase the depth of field, and improve the (electron) optical conditions in thick samples. The resolution achievable with a given acceleration voltage can be simply calculated by the following formula, assuming all points within a given thickness can be considered equally focussed:

1 $$d = \, \surd \left( {{2}\lambda t} \right)$$

where *d* is the resolution, *t* is the sample thickness (in this case, particle diameter), and λ is the electron wavelength^45^. For high symmetry specimens, such as icosahedral viruses, sample thickness (or depth) is functionally equivalent to particle diameter. Using Eq. (1), for a 250 nm thick sample, at an acceleration voltage of 300 kV, the electron wavelength is 1.97 pm, so the theoretical resolution limit is ~ 9.9 Å with the entire width of the particle at the same focal depth (black curves in Fig. 1). Extending this, at an accelerating voltage of 1 MV, the electron wavelength is 0.87 pm, so the theoretical resolution limit is calculated to improve to 6.6 Å.

Considering phase error in the contrast transfer function (CTF) of the signal^46^ is another estimation for calculating maximum attainable resolution (red curves in Fig. 1), and can be calculated by:

2 $${\text{d }} = \surd \left( { \, \left( {\lambda {\text{t}}} \right) / \left( {{2 } \times \, 0.{7}} \right) \, } \right)$$

In this case, the attainable resolutions are improved to 5.9 Å at 300 kV and 4.0 Å at 1 MV, respectively (red curves in Fig. 1). However, the value of 0.7 in Eq. (2)^46^ can vary based on the shape of an object, whether it is full or empty, and has not been tested on real objects such as tokyovirus (maximum diameter of 250 nm). Previous discussions^47–49^ cover the equations for calculating electron wavelengths and accounting for relativistic effects at given acceleration voltages if one wishes to further explore the mathematics. Of course, many other factors affect the maximum resolution achieved with experimental data. From the estimation, we attempted to clarify the structure of the entire giant virus particle with a maximum diameter of 250 nm at higher resolution using a 1 MV cryo-HVEM rather than using the block-based approach.

In this study, we first evaluated 1 MV cryo-HVEM for biological SPA. We then carried out SPA for a 250 nm maximum diameter icosahedral giant virus, tokyovirus. This resulted in a 7.7 Å three-dimensional (3D) reconstruction without using the block-based reconstruction technique. The cryo-EM map revealed a novel capsid protein network (Fig. 4). This consisted of MCP trimers and penton proteins covering the surface, an mCP layer consisting of eight protein components, and a scaffold protein component (ScPC) network. The penton protein is a unique pentameric protein located in the same layer as the MCP, but only at the fivefold vertices. The ScPCs existing underneath the mCP layer and connecting each vertex allows the internal membrane extrusion. As such, they may play a role similar to that of tape measure proteins that determined the particle size in NCLDV. An additional density on top of the MCP trimer suggested to include a glycoprotein, and which may function for host-cell interaction. Our results pave the way for a greater understanding of this family of NCLDV, and in addition provide the first evaluation of a new tool for the SPA of gigantic biological specimens.

## Results

### Performance of the 1 MV cryo-HVEM

**Figure 2:**
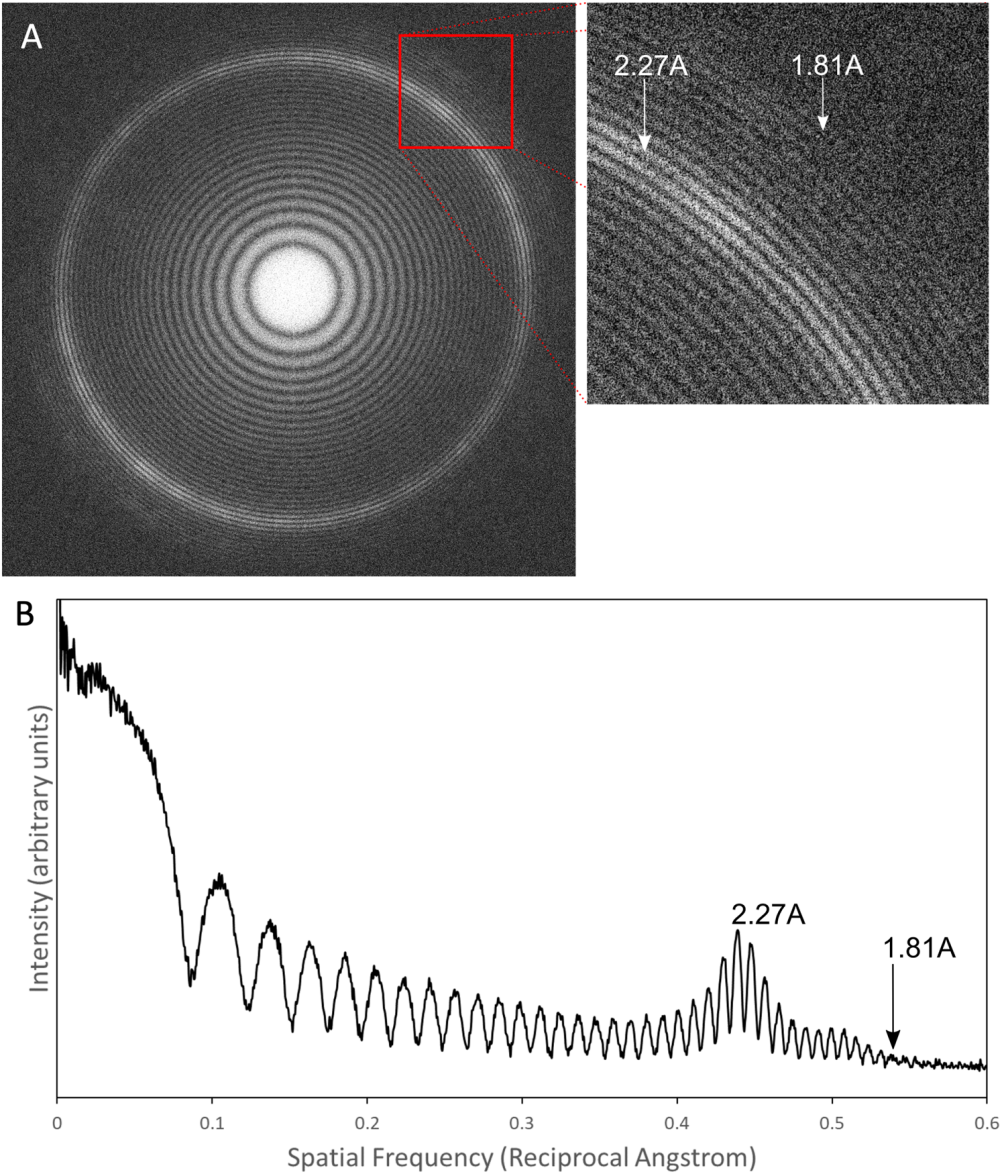
Performance of the cryo-HVEM (JEOL JEM-1000EES) equipped with a Gatan K2 Summit direct electron detector using a Pt-Ir standard sample. (**A**) Power spectrum showing clear Thon rings. (**B**) A focussed view of the Thon rings. (**C**) 1D plot of the power spectrum calculated with the "Radial Profile Extended" plugin of Fiji^75^.

While the imposed theoretical resolution limit is overcome by moving to higher acceleration voltages (Fig. 1)^45,46^, it is of little practical use if other factors are providing hard limits to attainable resolution. To this end, we examined the performance of the 1 MV cryo-HVEM (JEOL JEM-1000EES) using a Pt-Ir film and performed rotational averaging on a calculated power spectrum (Fig. 2A). In the 1D intensity plot, Thon rings are clearly visible to 1.81 Å (Fig. 2B). Comparing the performance of the 1 MV cryo-HVEM equipped with a LaB_6_ electron gun against a high performance 300 kV microscope using a thermal field emission electron gun (in this case a Titan Krios G2 (Thermo Fisher Scientific) was used) is also of great interest (Fig. S1). Both use Gatan K2 Summit direct detector as a camera. The Modulation Transfer Function (MTF) of the 1 MV microscope is superior in counting mode across the entire spatial frequency range compared to that of the 300 kV microscope, although in super resolution mode it drops significantly within the 0.25 Nyquist frequency (Fig. S1A). Detective Quantum Efficiency (DQE) is similarly superior for the 1 MV microscope in counting mode, but in super resolution mode it shows a significant reduction in both 1 MV and 300 kV microscopes (Fig. S1B). Furthermore, the DQE of the 1 MV cryo-HVEM show as much as a 40% drop in performance in the lower spatial frequencies compared to the 300 kV cryo-EM. Beyond 0.6 Nyquist frequency, the two are comparable. In this study, however, super resolution mode had to be used to increase the manual sampling efficiency of the large-size virus particles (Table 1).

**Table 1:** Cryo-EM data information.

**Tokyovirus data collection**			
Nominal magnification	15,000×		
(Corresponds to)	1.456 Å/pixel		
Microscope (accelerating voltage)	JEOL JEM-1000EES (1,000 kV)		
Spherical aberration (mm)	4.1		
Detector	Gatan K2 Summit		
Detector mode (dimensions in pixels)	Super resolution (7420 × 6780)		
Total exposure (e^−^/Å^2^)	~ 35		
Defocus target range	− 1.5 to − 4 μm		
Total frames per micrograph	160		
Total acquisition time per micrograph	32 s		
**Cryo-HVEM processing**			
Total selected particles	1529		
Final particles used	1182		
Pixel size	2.912 Å/pixel		
Symmetry imposed	I3		

Mask used	Capsid	Capsid + IM	Spherical
Map resolution (Å)	7.7	8.7	9.4
Map sharpening imposed (Å^2^)	− 100	− 100	− 100
EMDB accession code	30,797	30,798	30,799

### Overall structure of tokyovirus

**Figure 3:**
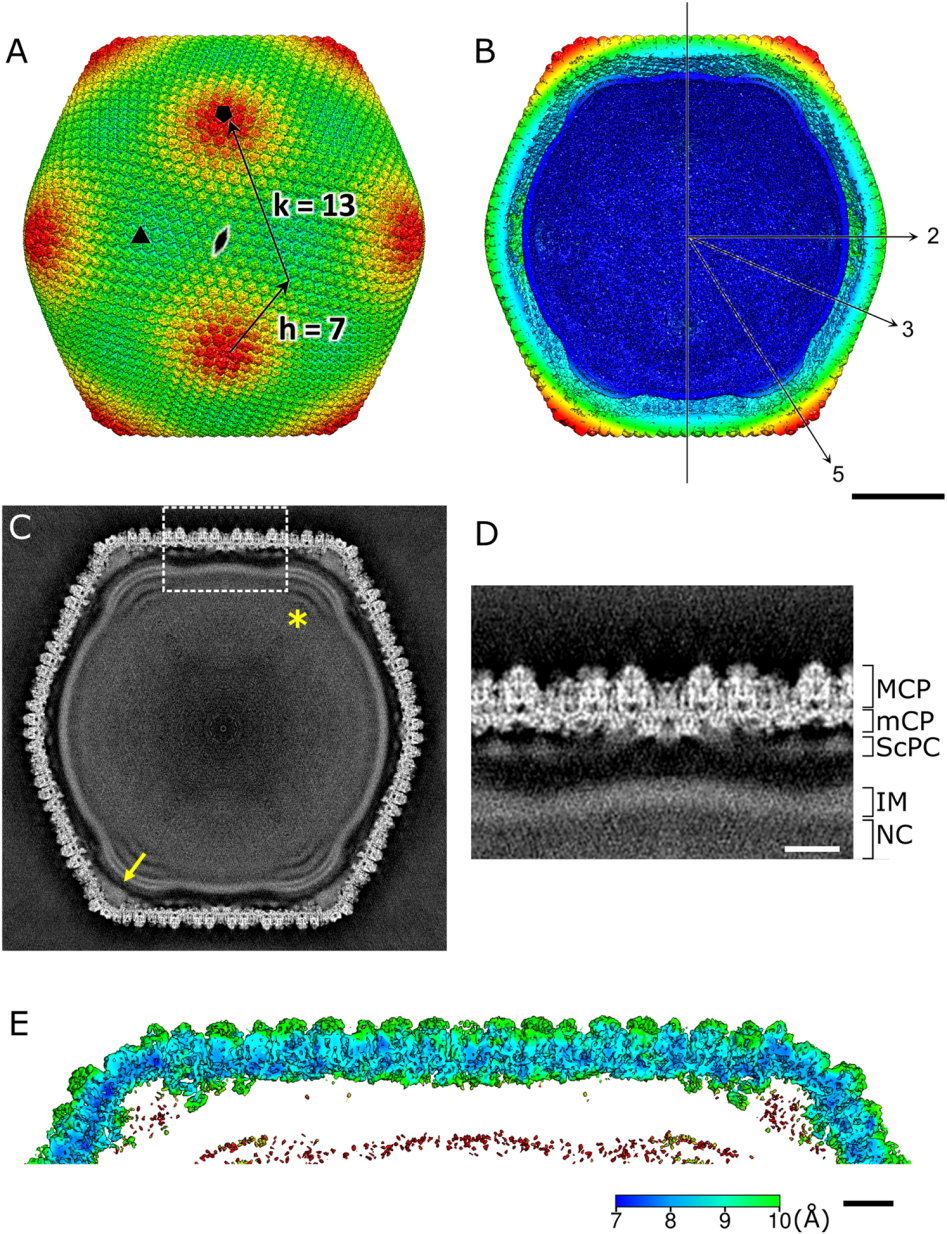
SPA 3D reconstruction of tokyovirus at 7.7 Å resolution. (**A**) An isosurface view, coloured by radius, and showing the fivefold (black pentagon), threefold (black triangle) and twofold (black double-teardrop) symmetry axes. H, K indexes indicating the T = 309 icosahedron are included. (**B**) A cross-section view, with symmetry axes shown with arrows. (**A**,**B**) are coloured by radius in UCSF Chimera with the following parameters: blue, 910 Å; turquoise, 1010 Å; green, 1080 Å; yellow, 1125 Å; red, 1200 Å and are shown at 2 σ. (**C**) A central slice of the 3D reconstruction. Asterisk shows a multilayer structure in the extrusion of the internal membrane. Arrow indicates weak densities connecting the vertex of the capsid and the internal membrane extrusion. (**D**) Focussed view of the marked box in (**C**), showing delineation between major capsid protein (MCP), minor capsid protein (mCP), scaffold protein component (ScPC), internal membrane (IM), and nucleocapsid (NC). (**E**) local resolution of the capsid, estimated by the *blocres* module of Bsoft^71^, focussing on the upper capsid edge, sliced to permit visualisation of internal density and is shown at 3 σ. Scale bars: (**A**–**C**) 50 nm, (**D**,**E**) 10 nm.

**Figure 4:**
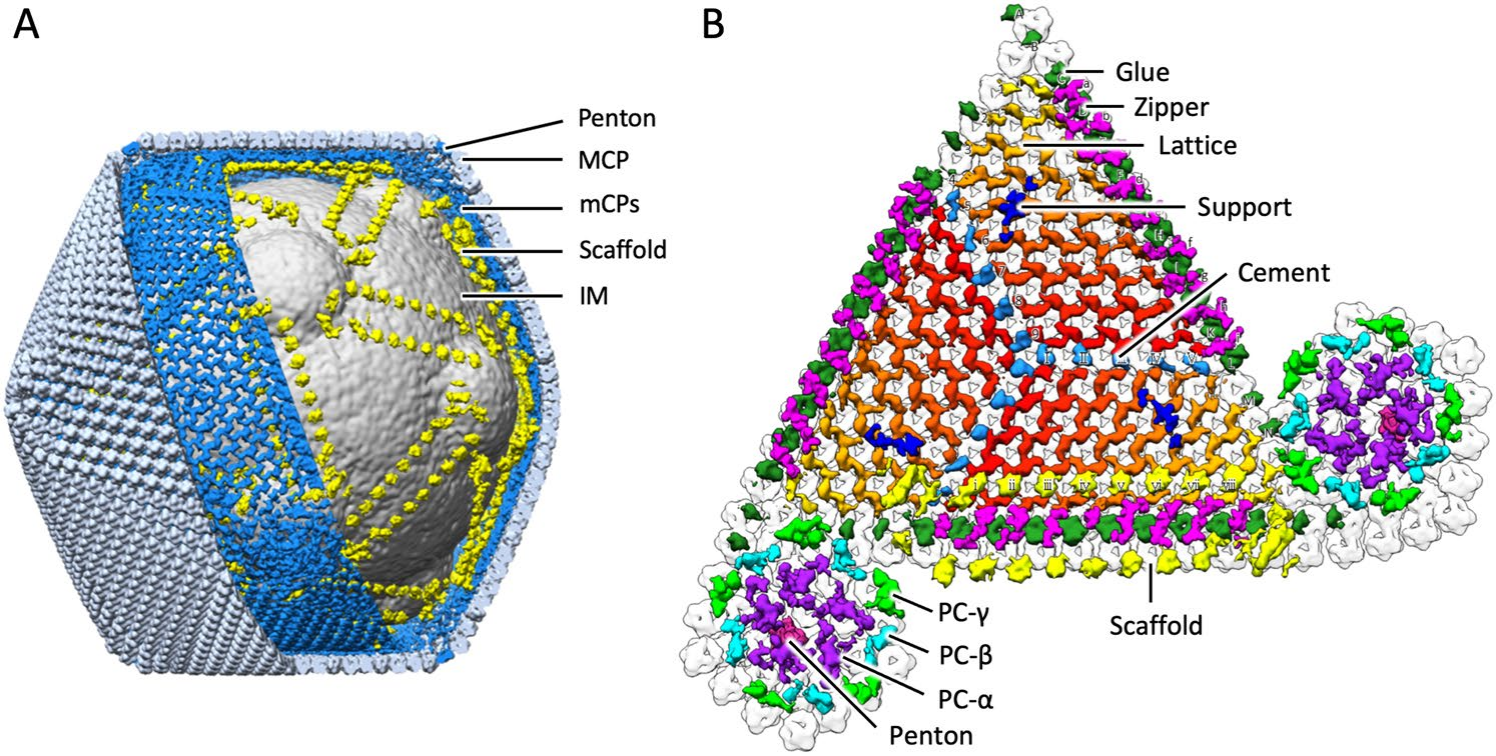
Segmentation of tokyovirus structure. (**A**) The complete tokyovirus virion cut out to show each component. Individual components of the virion; major capsid protein (MCP) layer (light blue), minor capsid protein (mCP) layer (blue), scaffold protein component (ScPC) array (yellow), and internal membrane (IM) (grey) are indicated. Individual segments are low-pass-filtered to improve visualisation clarity. (**B**) Focussed segmentation of mCPs, ScPCs (yellow), and penton (red purple) of tokyovirus. The mCPs, colored blue in (**A**), were classified into 8 components based on the structures, consisted of lattice component (LtC) (orange to red), support component (SuC) (royal blue), cement component (CmC) (sky blue), zipper component (ZpC) (pink), glue component (GlC) (emerald green), and 3 pentasymmetron components (PC-α, β, and γ) (purple, light green, and cyan). The isosurface is shown at 2 σ.

Figure 3 shows the overall structure of tokyovirus reconstructed using RELION 3.1^50^ with icosahedral symmetry imposed. Tokyovirus has an outer capsid shell comprised of MCP trimers arranged in an icosahedral form with T = 309 (h = 7, k = 13) (Fig. 3A). Immediately below the outer shell (MCP) are layers of mCPs and ScPCs in that order, and the internal membrane stores the viral nucleoid inside of these capsid layers (Fig. 3B–D). In the extrusion of the internal membrane, the membrane forms multiple layers (asterisk in Fig. 3C). Proximity to the vertex of the capsid from this extrusion is via the relatively large mCPs and low-density materials (arrow in Fig. 3C). Compared to MCP and mCPs, densities of ScPCs are smeared, showing structural flexibility of the component (Fig. 3C,D). Figure 3E shows the estimated local resolution of the capsid layer of tokyovirus, sliced for internal visualisation. The highest resolution was shown in the interface between MCP and mCPs. Each part has a characteristic structure, which can be better viewed by careful filtering and segmentation of the reconstruction (Fig. 4). MCP trimers cover the whole surface of the viral capsid except for the fivefold vertices (light blue in Fig. 4A), where the pentons plug the hole in the vertices (penton in Fig. 4A). mCPs can roughly be classified by eight distinct components, which we have temporarily named glue, zipper, lattice, cement, support, and pentasymmetron component (PC)-α, β, and γ (Fig. 4B). Further, the ScPCs are located underneath the mCPs (yellow in Fig. 4). These protein components can be consisted of several mCP proteins. At current resolution, and given current annotations of the genome, it is not possible to segment each protein or identify each protein according to the mass. We just identified these proteinaceous components by their shapes and locations. Unlike the other mCPs, the ScPCs form an anti-parallel chained array between pentasymmetrons along the trisymmetron interface (yellow in Fig. 4). Possibly as a result of the ScPCs frames, the internal membrane has a characteristic structure that extrudes outwards below the fivefold vertices (Fig. 3B,C, grey in Fig. 4).

### Minor capsid proteins (mCPs)

We segmented the tokyovirus reconstruction, and then extracted and classified the mCPs into eight types of protein components based on their structural features and arrangements (Fig. 4B). They are referred to as Lattice component (LtC) (orange to red in Fig. 4B), Support component (SuC) (royal blue in Fig. 4B), Cement component (CmC) (sky blue in Fig. 4B), Zipper component (ZpC) (pink in Fig. 4B), and Glue component (GlC) (emerald green in Fig. 4B), and three pentasymmetron components of α, β, and γ (PC-α, β, and γ) (purple, light green, and cyan in Fig. 4B), respectively. The mesh-shaped triangle formed by mCPs is composed of three trapezoidal units consisting of five components, LtC, SuC, ZpC, CmC, and GlC, and these trapezoids are connected by rotating 120° around the threefold rotation axes. These protein components are further connected to PC-β and PC-γ at the edge of the triangle.

**Figure 5:**
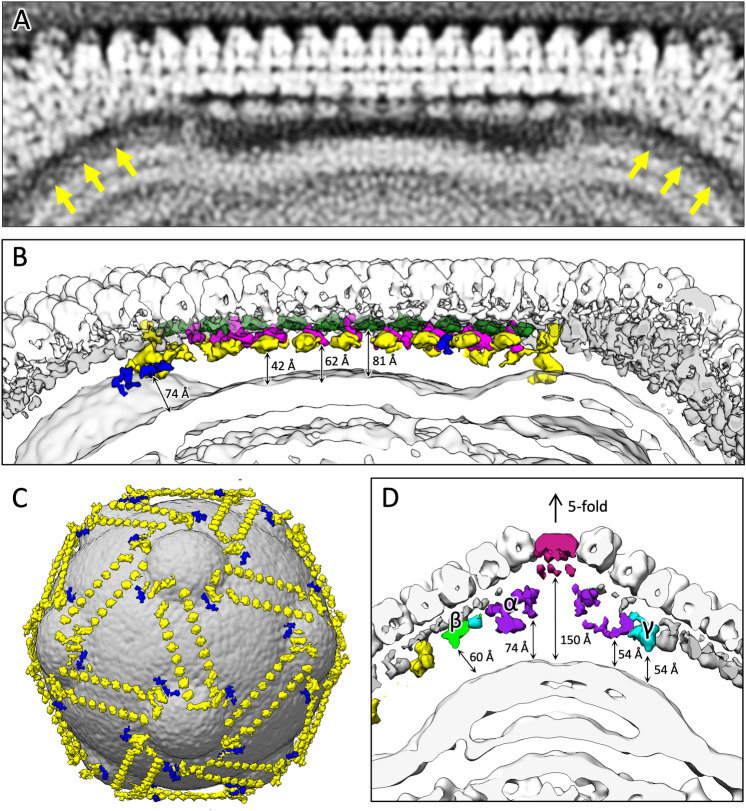
Examination of the scaffold protein component network between the internal membrane and capsid shell. (**A**) Slab density of the tokyovirus capsid along the trisymmetron interface showing the innermost layer of the mCP network. Arrows indicate weak densities connecting the vertex of the capsid and the internal membrane extrusion. (**B**) Isosurface view of the mCP components coloured as in Fig. 4 (GlC; green, ZpC; hot pink, ScPC; yellow, SuC; royal blue) and with distances from the internal membrane to the components indicated by arrows. (**C**) The tokyovirus internal membrane with ScPCs (yellow) and SuC (royal blue) network overlaid. (**D**) Focussing on the pentasymmetron at the fivefold axis, representing distances from the internal membrane extrusion to PC-α, β, γ (purple, light green, and cyan) and the penton (red purple). The isosurface is shown at 2 σ.

The LtC (orange to red in Fig. 4B) forms a wavy structure along the gap of MCP trimers (orange to red in Fig. 4B). This waveform repeats according to the number of MCP trimers present above each. It forms the base of the trapezoidal unit. Under the network structure formed by LtCs, the SuC (royal blue in Fig. 4B) creates a bridge between several LtCs (Fig. 4B). It also connects the two ScPC pairs (yellow in Fig. 5C) around the membrane extrusions, forming a three-dimensional ScPC framework surrounding the internal membrane (Fig. 5C). Within the trisymmetron, the CmCs (sky blue in Fig. 4B) connect the three trapezoidal units by interfacing the ends of LtCs, extending from the threefold axis toward the associated pentasymmetron and terminating at the ScPC edge. Two protein components, ZpC and GlC (pink and emerald green in Fig. 4B), are involved in connecting adjacent trisymmetrons and run directly above the ScPC array (Fig. 5A,B). The GlCs are present at the boundary between adjacent trisymmetrons and appears to glue them. The ZpCs fill the gaps between LtCs at the trisymmetron interfaces, interlocking with GlCs like the teeth of a zipper.

The ScPC is present further inside the mCP layer (yellow in Figs. 4, 5), forming a framework surrounding the internal membrane. ScPC has a large head and long tail structure, which is arranged along the edge of trisymmetron (Figs. 4 and 5B,C). Two ScPCs are connected in an anti-parallel manner with the head and tail, and both the heads are located at the apex of the pentagon of the pentasymmetron, surrounding the membrane extrusion (Fig. 5C). The adjacent ScPC terminal pairs are connected by SuC (royal blue in Fig. 5C).

### Penton and pentasymmetron components

The penton density (central region in Fig. S2A) is similar to that of PBCV-1 (Fig. S2B), in that it has a “cap” region which aligns with the MCP layer and a lower region which aligns with the mCP layer. ASFV, by comparison, is missing this lower region (Fig. S2C). We fitted the PDB model of the PBCV-1 penton (Fig. S2E) to the upper region of the tokyovirus penton (Fig. S2D), showing the tokyovirus penton has the similar volume and structure as the PBCV-1 penton, though the homologous protein of the PBCV-1 penton has not been identified in tokyovirus. In ASFV, the insertion domain is extrinsic of the MCP interface (Bracket in Fig. S2F), while neither tokyovirus nor PBCV-1 exhibit clear density for this domain. This insertion domain appears in ASFV. It may function to assist insertion of DNA into the capsid, as ASFV also has an external membrane to penetrate^16^. This domain is also present in Cafeteriavirus-depedent mavirus (a virus which infects a specific NCLDV)^51^. The penton density including the lower region itself has a distance to the internal membrane extrusion of ~ 150 Å (Fig. 5D). The gap was supported with three pentasymmetron components (PC-α, β, and γ) and lower density materials (arrow in Fig. 3C).

The three pentasymmetron components of PC-α, β, and γ keep a similar distance from the internal membrane extrusion (54–60 Å) (Fig. 5D), which is also similar to that of the ScPC array along the trisymmetron interface (42 Å) (yellow in Fig. 5B). PC-α (purple in Figs. 4B and 5D) is the largest, interacting with each other and the penton itself (Figs. 4B and 5D). Pairs of PC-β and PC-γ (light green and cyan in Figs. 4B and 5D) surround this group of PC-α. PC-β interacts primarily with a single PC-α, while also acting as terminal for the GlC (Fig. 4B). PC- γ interacts with two neighbouring copies of PC-α. These protein components function to support the MCP array in the pentasymmetron. They also maintain contact with the mCP network under the trisymmetron and interact with the extrusion of the internal membrane via poorly resolved low density materials under the fivefold vertices.

### Internal membrane and its interaction with ScPCs

Melbournevirus, one of *Marseilleviridae*, was first reported to possess a very characteristic extrusion of the internal membrane at the fivefold axes^28^. Tokyovirus also shows this extrusion of the internal membrane, and further, permitted identification of multiple layers in the extrusion (asterisk in Figs. 3C and 5A,B,D). Interestingly, the large heads of the ScPC surround the membrane extrusion together with SuCs (Fig. 5C). The ScPC array and SuCs may play a role in distorting the membrane into this extruded shape at the fivefold axes.

### Major capsid protein

A BLAST search^52^ was performed using the proposed MCP sequence from the draft genome of tokyovirus^29^ (Table 2). The MCP of tokyovirus shows the highest scores against iridovirus (PDBID: 6OJN)^14^ and PBCV-1 (PDBID: 5TIP)^53^, with high coverage and moderate homology, although both comparisons of conserved sequences fall below the "identities" that allows for direct and safe comparisons^54^. The three respective MCP amino acid sequences were aligned by PROMALS3D^55^. The secondary structure of tokyovirus MCP was independently predicted by PSIPRED^56^, which identified two sets of eight β strands (B1 to I2 in Fig. S3) that form a (double) jelly roll motif like the MCP of other NCLDVs^7^.

**Table 2:** Results of BLAST comparison of tokyovirus MCP versus other NCLDVs.

Virus	PDB ID	a.a.		BLAST search		
			E-value	Score	Query cover (%)	Identities (%)
Iridovirus	6OJN	463	1.00E−105	310	99	38.21
PBCV-1	5TIP	436	5.00E−17	68.9	96	19.92
Faustovirus	5J7O	645	0.016	23.9	30	37.5
Phage PM2	2VVF	269	0.043	21.2	14	26.67
ASFV	6KU9	693	0.069	21.9	38	12
Sputnik virus	3J26	508	0.48	18.9	27	36
PRD-1	1CJD	394	0.93	17.3	16	37.04
Vaccinia virus	2YGB	569	2.4	16.5	14	29.63
Phage FLiP	5OAC	310	2.7	15.4	15	42.86
Adenovirus	6B1T	952	3	16.9	16	21.43
STIV	2BBD	350	4.4	15	13	22.73
Mavirus	6G45	610	4.8	15.8	12	25.93

**Figure 6:**
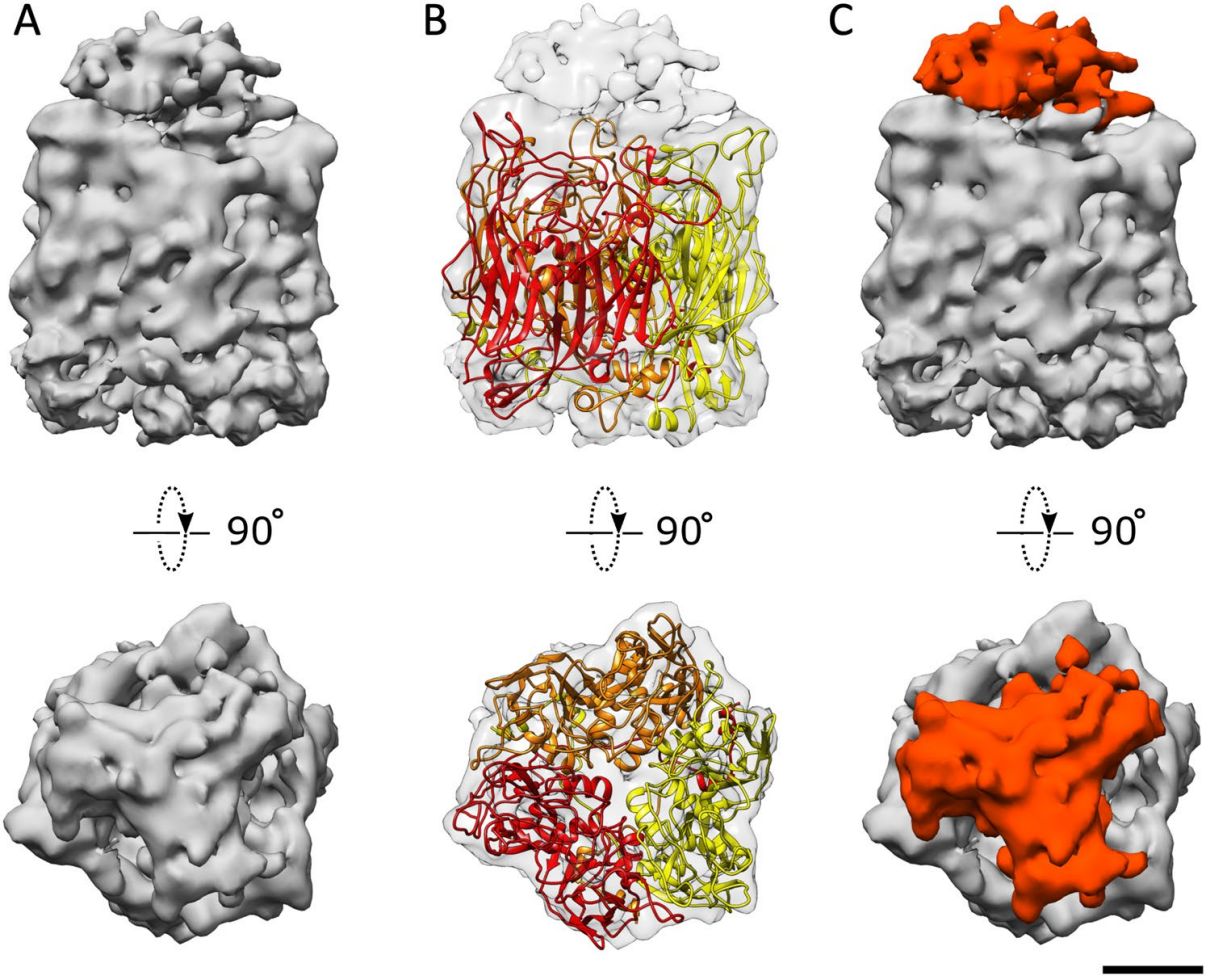
Cryo-EM map and fitted homology model of the tokyovirus MCP trimer. The homology model was generated by SWISS-MODEL^57^ and adjusted to the volume with COOT^58^ and refined with PHENIX^59^. (**A**) Side and top views of the extracted cryo-EM map of the MCP trimer. (**B**) Side and top views of the extracted MCP trimer cryo-EM map with the homology model fitted. (**C**) Side and top views of the extracted MCP cryo-EM map of the MCP trimer with the additional cap region coloured in red. Scale bar equals 2 nm. The isosurface of the extracted MCP segment is shown at 2 σ.

In the MCP amino acid sequence of each virus, there is a large difference in loop length between β strands (Fig. S4). To investigate how the difference in loop length appears in the difference in MCP structure, a homology model of tokyovirus MCP was generated based on the MCP model of PBCV-1 using the SWISSMODEL server^57^. The MCP trimer from a threefold axis of the tokyovirus cryo-EM map was extracted (Fig. 6A). Then, the homology model was manually optimised to fit the MCP density using COOT^58^ and energetically minimized by PHENIX^59^ to ensure that the structure is valid (Fig. 6B). The resultant homology model was compared to both MCP models of PBCV-1 and iridovirus (Fig. S4). In tokyovirus MCP, the loops connecting the external parts of jelly roll 1 (JR1) or JR2 are longer than those of PBCV-1. The DE1 and FG1 loops are longer than those of PBCV-1 and of similar length to those of iridovirus. The HI1 loop is particularly extended (Figs. S3 and S4), with the 23-residue sequence of tokyovirus being longer than that of PBCV-1. Conversely, the DE2 loop in tokyovirus is truncated compared to those of both PBCV-1 and iridovirus. Other loops are of comparable length. These loops constituting the external part of the MCP model coincide with the external part of the density of the tokyovirus MCP trimer, but do not fill the entire cap density of the tokyovirus MCP trimer (Fig. 6B). The density in the tokyovirus MCP which is not filled by the fitted homology model has been highlighted in red (Fig. 6C).

The MCP density has a visible cap (red in Fig. 6C) which does not correspond to any part of the fitted homology model. To check this, we extracted three MCPs from different areas of the capsid—one from the threefold axis itself, one from two MCP trimers away from the threefold axis, and one from an intermediate point between the two-, three- and fivefold axes. Direct cross correlation between these ranged from 0.99 to 0.91 when calculated in UCSF Chimera^60^. All MCP trimers possess this cap density. A previous report^28^ for Marseilleviruses showed that a small protein indicated by proteomic analysis was present at approximately the same levels as the MCP protein. However, due to the nature of SPA (that of averaging many particles) and imposition of icosahedral symmetry, it is possible that not all MCPs are fully occupied. The correlation of the cap density ranges from 0.98 (the threefold cap density against the symmetrised threefold cap density) to 0.87 and 0.84 for the other two MCP positions, respectively. This lower correlation for the cap density may be caused by higher flexibility, which is likely if the protein is highly glycosylated. The symmetric nature of this cap implies the presence of protein rather than disordered post-translational glycosylation of the MCP. This is further supported by Periodic acid Schiff (PAS) staining^61^ of SDS-PAGE-separated tokyovirus proteins, which do not show any glycosylated proteins at the molecular weight of the MCP. However, a band is evident at ~ 14 kDa (Fig. S5), indicating that the cap density is likely to include a protein which has been glycosylated interacting with the top of the MCP.

## Discussion

Here we applied 1 MV cryo-HVEM to the structural analysis of tokyovirus as a complete viral particle, overcoming some of the resolution limits imposed by the effect of depth of field on exceptionally large particles. The 3D reconstruction showed the highest resolution of a giant virus larger than 200 nm without utilising the block-based reconstruction technique^38^ or similar methods^16^. However, the current resolution of 7.7 Å of tokyovirus has not reached the theoretical maximum of 4.0 Å for ~ 250 nm particles (red curve in Fig. 1). In practice, many factors affect the ability to achieve a given resolution beyond the accelerating voltage. Therefore, before studying the biological specimen, the performance of the microscope system was confirmed with Thon rings of a Pt-Ir standard film extending beyond 2 Å (Fig. 2). This demonstrates that the microscope or electron source would not be a limiting factor in resolutions achieved with the giant virus particles, even though it does not have a field emission electron source and is using a less coherent LaB_6_ source.

Although there were many factors that limited the resolution of tokyovirus to 7.7 Å, the main limiting factor for current resolution is likely the amount of data that can be collected. Cryo-HVEM requires the manual collection of micrographs. The 1182 particles from 160 micrographs used in total were an extremely small number compared to other reported SPA of giant viruses using 300 kV microscopes. PBCV-1 with a maximum diameter of 190 nm was reconstructed with ~ 13,000 particles from 5624 images at 4.4 Å reconstruction^15^ and ASFV with a 250 nm maximum diameter of the outer capsid were reconstructed with 16,266 particles from 17,135 micrographs at 14.1 Å reconstruction^62^, and with 63,348 particles from 64,852 micrographs at 8.8 Å reconstruction^16^. The size of the virus imposes limits which require careful balance between a lower magnification to increase particles imaged per micrograph and a high enough magnification to achieve worthwhile resolutions in reconstructions. Tokyovirus with a maximum diameter of 250 nm is so large that even at relatively low magnifications, there were at best around 12 virus particles per micrograph (often ~ 6 virus particles, e.g., in Fig. S6A) which can be utilised for 2D classification. In this case, the “super resolution” mode (7420 × 6780 detector dimensions) in the K2 Summit camera was used for data acquisition, keeping the pixel spacing less than 1.5 Å/pixel (Table 1). Automated acquisition of micrographs, which is now near ubiquitous in the mainstream cryo-EM would greatly increase the quantity of data which can be acquired in any given timeframe. However, we selected “super resolution” mode for manual-operated cryo-HVEM to maintain a better particle count (per micrograph) while maintaining a higher sampling frequency, though the detector performance suffers slightly (Fig. S1). This probably imposes further limitation on the attainable resolution in this experiment.

Another limitation on the attainable resolution lies in the software. Some elements of general-purpose cryo-EM SPA image processing do not support HVEM accelerating voltages. Gctf^63^ was not used as it does not support 1 MV data, and likewise, dose weighting of 1 MV data is unsupported in both MotionCor2^64^ and the RELION implementation^65^ as there are currently no parameters for radiation damage at 1 MV. Fortunately, CTFFIND^66^ does support 1 MV data for contrast transfer function (CTF) estimation, so we used it in this study. We were unable to use dose weighting, although this is somewhat offset by higher acceleration voltages, which reduces radical damage^67^. Particle polishing in RELION^65^ also provides little benefit, which may be caused by the large size of the viruses on the micrograph where distortions across the micrograph potentially cause virus deformation variation per frame. Ewald sphere correction of whole particles, recently implemented in the RELION software suite^65^, has also shown to improve reconstructions from 300 kV microscopes across a range of diameters. We tested Ewald sphere correction for our tokyovirus reconstruction taken with the 1 MV cryo-HVEM and found no improvement. This may be because the 7.7 Å reconstruction of tokyovirus is not high enough resolution to benefit. The software support mentioned above, particularly dose weighting, will be needed for high resolution SPA of giant viruses using 1 MV microscopes in the future.

Ultimately, using cryo-HVEM provides benefits for larger particles. The decreased defocus gradient across a particle aids high resolution whole particle reconstruction. Sample damage from the electron beam is also decreased, which is of particular importance with biological samples. Furthermore, increased beam penetration permits improved visualisation of internal structure, as used in cryo-electron tomography with pithovirus, an amphora-shaped giant virus having ~ 800 nm thickness^8^. Beyond the more widely available 300 kV cryo-EM, it permits study of finer details of even larger viruses, which lower acceleration voltages are unable to penetrate. Finally, the reconstruction methodology is simpler than that of block-based reconstruction. Block-based reconstruction is a powerful technique, but as the name implies, it breaks the whole structure down into blocks. Stitching these back together is called a composite map, and while these are gaining in popularity, do have some inherent dangers. For example, FSC calculations cannot be performed on composite maps without artefacting, and sharpening can reveal overlap artefacts between maps. We tested block-based reconstruction on data sets from cryo-HVEM, but it did not improve attained resolution. Although the exact reasons are unknown, our working hypothesis is that the relatively low number of particles in the final reconstruction and the lower curvature of the Ewald sphere at 1 MV (when compared to 300 kV) decreases the effectiveness of block-based reconstruction. However, block-based reconstruction methods may have some advantages in cryo-HVEM given sufficient particle numbers. This is because it can overcome minor distortions of very large symmetric particles, yielding improved resolutions. For objects with low symmetry the direct method using higher accelerating electrons is still needed.

The 7.7 Å capsid structure of tokyovirus represents a new capsid network of NCLDV. The trapezoidal arrays of LtCs in tokyovirus do not form a single underlayer beneath the MCP, like the mCPs of PBCV-1^15^ or ASFV^16^, instead relying on another CmC which connect the three rotated trapezoids within the trisymmetron (Figs. 5, S7). Two protein components of GlC and ZpC are present at the trisymmetron interfaces rather than a single protein (P11) as in PBCV-1. SuC also interacts with each trapezoidal lattice on the interior surface, and further forms a connection with ScPCs, which does not appear to be present in the reconstructions from PBCV-1 and ASFV.

The T = 309 (*h* = 7, *k* = 13) capsid is clearly displayed in the 3D reconstruction (Fig. 3). With this structure we can newly identify an intermediate “scaffold” protein component (ScPC) array between the capsid and the internal membrane (yellow in Figs. 4 and 5). Anti-parallel pairs of ScPCs run along the trisymmetron interface and connects with each pentasymmetron via the pentasymmetron components. These features are quite analogous to the “tape-measure” proteins^40^ reported in PBCV-1 and ASFV cryo-EM maps^15,16^, which is the long filamentous mCP named P2 in PBCV-1 and M1249L in ASFV. This suggests that tokyovirus capsid construction may occurs through a different mechanism. The presence of this scaffold network may indicate that the scaffold is responsible for imposing restraints on the capsid dimensions. Greater clarity of these flexible ScPCs will be required to further elucidate their interactions in more detail and perhaps shed more light on their potential role in construction.

To build the MCP homology model, we extracted the central MCP trimer from a trisymmetron and the homology model was rigid-body-fitted into the density. Regions of the homology model which fit the density poorly were manually fixed using COOT^58^ and energetically refined with PHENIX^59^ software (Fig. 6B). An empty “cap” region was present on each MCP trimer density when the MCP homology model was fitted to it, although thus far we have been unable to clarify this region sufficiently for model fitting. As such, we were initially unsure whether this density is caused by other smaller proteins interacting with the MCP or post-translational modification of the MCP. We identified a candidate of the small “cap” protein, as Periodic acid Schiff (PAS) staining^61^ was used on SDS-PAGE of purified tokyovirus particles to identify potential glycoproteins. This showed a protein running at ~ 14 kDa (Fig. S5B). However, the band is weak compared to that of MCP in the Coomassie stained gel (Fig. S5A). The cap density may not be present for all MCP trimers, so called “partial occupancy” and the icosahedral averaging will affect their clarity as a result. In the case of PBCV-1, sugar chains are directly bound to the MCP^53^, while in the case of ASFV, there is a second membrane external of the capsid itself^16^ so additional capsid glycosylation is less important. We previously found that some species in the *Marseilleviridae* family induce a bunch formation in host amoeba^20^. Simultaneously, the newly borne viruses adhered to and aligned on the host cell surface in the process. A member of the *Mimiviridae*, tupanvirus, has also evidence suggesting this similar mechanism, where a mannose-binding protein (MBP) expressed on the amoeba cell membrane was suggested to be involved in intercellular adhesions^68^. In Marseilleviruses, 10 of the 49 identified virion proteins were reported to be glycosylated^18^. The glycoprotein may play a role in these bunch formation and adhesion to the cell, potentially to increase speed of transmission, but it needs further study to clarify the precise function.

The tokyovirus and PBCV-1 pentons are similar in depth (Fig. S2A,B). As the tokyovirus penton density is fitted with that of the cryo-EM-derived PDB model of PBCV-1 (PDBID: 6NCL) (Fig. S2D), we can find a density present for the penton base protein and an unmodeled lower region level with the mCP layer. The same is evident in PBCV-1 (Fig. S2E). The ASFV penton protein (Fig. S2F) fits the single jelly roll in the same position and orientation as the PBCV-1 penton, with the insertion domain on the outer face of the capsid in density, for which neither PBCV-1 nor tokyovirus possess clear density. A BLAST search of the tokyovirus genome, using both the PBCV-1 penton and the Cafeteriavirus-dependent mavirus penton which was modelled into the ASFV penton structure, did not yield any matches, so identification and homology modelling of the penton protein has not been possible. Given the low BLAST metrics for the tokyovirus MCP against both PBCV-1 and mavirus (Table 2) this is not necessarily surprising. To clarify the nature of the protein occupying the lower region of the penton, we must achieve a higher resolution reconstruction.

Immediately around this small pentamer is a symmetric array of further protein components of PC-α, β and γ within the mCP layer, which act to support the MCP array in the pentasymmetron as a replacement for the lattice proteins and interact with the internal membrane extrusion via low-density materials (arrows in Figs. 3C and 5A). They may be roughly analogous to the “lantern” proteins in ASFV^16^. These further extend to the edge of the pentasymmetron and interact with the ScPC array (Fig. 4B). This is a novel structure in NCLDV and likely to play a role in the formation of the internal membrane extrusion.

In conclusion, based on the theoretical resolution estimation, we applied 1 MV cryo-HVEM to single particle analysis of tokyovirus, which is a large virus particle with a maximum diameter of 250 nm. This revealed the 7.7 Å capsid structure with a limited quantity of data without using a block-based reconstruction technique. The cryo-EM map showed the novel capsid network allowing the characteristic internal membrane extrusions under the icosahedral vertices. The advanced ScPC array represents a framework that supports the extruded structure of the internal membrane and also possibly function as a tape measure protein reported in other NCLDVs. The unique PC-α, β, and γ are also likely to support the membrane extrusions. While it shares many similarities to the structures of PBCV-1 and ASFV regarding MCP structure, we found a cap density on top of the MCP that was suggested to contain a glycoprotein. The glycoprotein identified in *Marseilleviridae* possibly acts to interact with the host cell surface. This in turn may cause the bunch formation of host cells in some species as has been previously demonstrated^20^. The 1 MV cryo-HVEM installed in Osaka University had sufficient performance to reach the theoretical resolution, but the current resolution of tokyovirus capsid was limited to 7.7 Å. One of the major problems was with the limited quantity of data. This will be solved in the future by installing automated data acquisition software. Using a larger format detector or improving the super resolution performance of the Gatan K2 detector when paired with HVEM will also remedy the problem, because the significant drop in low frequency signal in super resolution mode at 1 MV may be a limitation of the super resolution estimation algorithm for higher energy electrons in the current K2 detector (Fig. S1). Another major problem is the limited support of software for 1 MV micrographs. If current advanced techniques such as dose weighting during motion correction, and Bayesian particle polishing can be used, and full CTF parameter refinement can be optimized, the combination of all of these should allow the resolution to approach the theoretical value. Further optimizations and detailed analytical evaluations to solve the problems identified in this study will be necessary for cryo-HVEM using a LaB6 electron source to be used for structural analysis of large biological samples. To distinguish the resolution limitations of cryo-HVEM, we should also test SPA using well-tested specimens, such as apoferritin, TMV, and/or GroEL. In addition, careful comparisons with mainstream 300 kV cryo-EM would help demonstrate the potential of cryo-HVEM in the future.

## Methods

### Tokyovirus growth, purification, and sample preparation

Tokyovirus^29^ was originally provided by Professor Masaharu Takemura, Tokyo University of Science. It was propagated in *Acanthamoeba castellanii* cells cultured in PYG medium (2% w/v proteose peptone, 0.1% w/v yeast extract, 4 mM MgSO_4_, 0.4 mM CaCl_2_, 0.05 mM Fe(NH_4_)_2_(SO_4_)_2_, 2.5 mM Na_2_HPO_4_, 2.5 mM KH_2_PO_4_, 100 mM sucrose, pH 6.5). Viruses were purified as described previously^27,28^. Summarily, the infected culture fluid was collected and centrifuged for 10 min at 1500*g*, 4 °C to remove dead cells, before the supernatant was centrifuged for 35 min at 10,000*g*, 4 °C. The pellet was suspended in 1 ml of PBS buffer and loaded onto a 10–60% sucrose gradient, before further centrifugation for 90 min at 8000*g*, 4 °C. The concentrated band was extracted and dialysed in PBS before a further round of centrifugation with the same conditions. The pellet was suspended in PBS before cryo-EM grids were prepared.

### SDS-PAGE and periodic acid schiff staining

A sample of the purified tokyovirus was treated with SDS-PAGE sample buffer (2332330/AE-1430 EzApply, ATTO) and boiled for 5 min. 10 μg of each denatured sample was loaded into a well (2331635/CHR12.5L c, ATTO), and 7 μl of standard molecular marker (2332340/AE-1440, ATTO) run to estimate the molecular weights of the resulting protein bands. Electrophoresis was performed at constant current of 10.5 mA with an electrophoresis buffer (2332323/WSE-7055, ATTO) using an Electrophoresis system (2322240/WSE-1010, ATTO). The resultant gel was stained with a regent (2332370/AE-1340, ATTO). Periodic acid Schiff staining was carried out according to the protocol described in the PAS stain kit (GlycoGel Stain Kit 24693-1, PSI).

### Performance test of 1 MV cryo-HVEM

A JEOL JEM-1000EES cryo-HVEM (JEOL Inc.) installed at the research center for ultra-HVEM of Osaka University was used. The microscope was equipped with a LaB_6_ filament electron gun at an accelerating voltage of 1 MV, an autoloader stage which can keep up to 12 frozen-hydrated EM grids at cryogenic conditions, and K2 IS direct detector camera (Gatan Inc.). The stage and storage of the sample was always cooled with liquid nitrogen that is automatically replenished. Image data were collected manually. For resolution limit test of the 1 MV cryo-HVEM, Pt-Ir film (JEOL Inc.) was imaged at a nominal magnification of 100,000 × (0.22 Å/pixel on specimen) and 0.6–2 μm defocus. Movie images were recorded on K2 IS camera in counting mode at a dose rate of ∼ 8 e^−^/pixel/s with 0.2 s/frame for 1 s exposure. Motion-corrected full frames were summed with DigitalMicrograph software (Gatan Inc). For image quality test, MTF and DQE curves were measured using the shadow of a beam stopper metal blade in both counting mode and super-resolution mode of the Gatan K2 IS in the cryo-HVEM. The data was processed with FindDQE^69^. For comparison, the data was collected with the same conditions using a 300 kV Titan Krios G2 (Thermo Fisher Scientific) and K2 Summit camera installed in the institute.

### Cryo-HVEM data acquisition of tokyovirus

An aliquot (2.5 μL) of the purified tokyovirus particles was placed onto R 1.2/1.3 Quantifoil grids (Quantifoil Micro Tools) that were glow-discharged using a plasma ion bombarder (PIB-10, Vacuum Device Inc.) immediately beforehand. This grid was then blotted (blot time: 10 s, blot force: 10) and plunge-frozen using a Vitrobot Mark IV (Thermo Fisher Scientific) with the setting of 95% humidity and 4 °C. A total of 304 micrographs were manually collected using a JEOL JEM-1000EES (JEOL Inc.) equipped with an autoloader stage and K2 Summit camera (Gatan Inc.) optimised for 1 MV HVEM in a total of seven sessions using three grids. Micrograph movie frames were collected in super resolution mode at a magnification equivalent to 1.456 Å/pixel with a target defocus of 2–4 μm. Each exposure was 32 s, at a frame interval of 0.2 s for a total of 160 frames per micrograph. The frames were stacked using EMAN2^70^.

### Image processing of tokyovirus

Micrograph movies were imported into a beta build of RELION 3.1, before motion correction with the RELION implementation^65^ of MotionCor2^64^. Motion correction was performed using 5 × 5 patches with B-factor blurring of 500 Å^2^. Contrast Transfer Function (CTF) estimation of the images was carried out using CTFFIND 4.1.10^66^ with the following parameters: lower defocus limit, 2500 Å; upper defocus limit, 80,000 Å; step size, 100; exhaustive search. After the first run using a box size of 512, good CTF fits were selected, and failed fits were re-run using a larger FFT box size. This was repeated until an FFT box size of 2,048. Other parameters were left at default settings. After this point, micrographs which failed to find a good fit were discarded. We define a “good” CTF fit as one which has distinguishable Thon rings and with which the simulated power spectrum aligns. This resulted in a total of 156 micrographs being carried forward. 1529 particles were manually picked, extracted with 4× downsampling to 5.824 Å/pixel (2400 × 2400 pixel boxes become 600 × 600 pixel boxes) and 2D classified into 40 classes with a circular mask diameter of 2600 Å, angular sampling of 2° (automatically increased to 3.75° due to use of GPU acceleration) and a search range of 7 pixels. 1458 particles were carried over in good classes, and 2D classified a second time with 1° angular sampling (automatically increased to 1.825°) and “ignore CTF until first peak” enabled, resulting in 1419 particles in good classes. All 3D processing was carried out with icosahedral symmetry imposed. An initial model was generated with the stochastic gradient descent algorithm in RELION 3.1. These particles were passed to 3D classification into 5 classes, using 1.8° angular sampling for 25 iterations, followed by 0.5° angular sampling for a further 25 iterations. The two best classes were selected, totalling 1,297 particles. 3D refinement was carried out using only a spherical mask. Particles were re-extracted and re-centred with 3× downsampling (4.368 Å/pixel) and refined further, then re-extracted and re-centred with 2× downsampling (2.912 Å/pixel). Magnification anisotropy refinement and CTF refinement were carried out, improving attained resolution. 3D classification into 5 classes with alignment disabled was carried out using a mask blocking the disordered internal volume (viral DNA) and the best class selected comprising 1182 particles. Particle polishing had a negligible effect when used. Final post-process resolution depends heavily on the mask used: a capsid-only mask results in a 7.7 Å estimated resolution, including the scaffold proteins and internal membrane lowers this to 8.7 Å. A soft spherical mask gives a 9.4 Å resolution. Local resolution was calculated using the *blocres* module of Bsoft^71^ with no mask applied. The full pathway through the SPA 3D reconstruction is summarized in Fig. S6.

### Bioinformatics, model building and fitting of MCP

The MCP protein of tokyovirus was identified from the previously reported genome. The primary amino acid sequence of tokyovirus MCP was compared to those of other NCLDVs by BLASTP^72^, and the SWISS-MODEL^57^ and I-TASSER^73^ servers were used to generate a homology model using the MCP model of PBCV-1 (PDBID: 5TIP)^53^ as a template. This homology model was rigid body fit into a tokyovirus MCP trimer extracted from the threefold symmetry axis and sections of the model which lay outside of density were adjusted by COOT^58^ and energetically refined by PHENIX^59^. The central point of the trisymmetron was identified visually and extracted using Volume Eraser tool in UCSF Chimera^60^, then the volume was cropped to 100^3^ pixels. The resulting model was saved. The trisymmetron map was segmented using the SEGGER^74^ module of USCF Chimera and segments which overlapped the model saved into a separate volume. For the penton base protein, the PDB structure of the penton of PBCV-1^15^ was fitted to the centre of the fivefold axis and SEGGER was used to extract the region. This also selected the majority of the adjacent five MCPs. Therefore, *molmap* in UCSF Chimera^60^ was used to generate a 15 Å molecular map, and the map was passed to the *relion_mask_create* module of RELION 3.1 to generate a binary mask with an extension of 5 pixels and a soft edge of 5 pixels. The mask was then imposed on the extracted region.

### Visualisation of data

The 3D reconstructions of tokyovirus were visualised using *relion_display* module of RELION 3.1^50^ or UCSF Chimera^60^ depending on dimensionality. Pt-Ir power spectra were visualised using Fiji^75^ and the rotational averages were calculated using the Radial Profile Extended plugin.

## Supplementary Information

Supplementary Figures.

## Data Availability

The cryo-HVEM reconstructions have been uploaded to the EMDB and are available at the following accession codes, 30797: capsid-only post-process mask, 7.7 Å, 30798: capsid, scaffold and internal membrane post-process mask, 8.7 Å, 30799; soft spherical post-process mask, 9.4 Å.
