# Supplementary material for: A novel capsid protein network allows the characteristic internal membrane structure of Marseilleviridae giant viruses
Source: Sci Rep. 2022 Dec 11;12:21428. doi: 10.1038/s41598-022-24651-2 (PMC9742146; doi:10.1038/s41598-022-24651-2)
Supplement: Supplementary file 1 — Supplementary Figures. [file 41598_2022_24651_MOESM1_ESM.pdf]

Supplementary Information

A novel capsid protein network allows the characteristic internal membrane structure of *Marseilleviridae* giant viruses

Akane Chihara[1,2,3,6], Raymond N. Burton-Smith[2,3,6], Naoko Kajimura[4], Kaoru Mitsuoka[4], Kenta Okamoto[5], Chihong Song[1,2,3*], Kazuyoshi Murata[1,2,3*]

[1] Department of Physiological Sciences, School of Life Science, The Graduate University for Advanced Studies (SOKENDAI), Okazaki, Aichi, Japan

[2] Exploratory Research Center on Life and Living Systems (ExCELLS), National Institutes of Natural Sciences, Okazaki, Aichi, Japan

[3] National Institute for Physiological Sciences, National Institutes of Natural Sciences, Okazaki, Aichi, Japan

[4] Research Center for Ultra-High Voltage Electron Microscopy, Osaka University, Ibaraki, Osaka, Japan

[5] Program in Molecular Biophysics, Department of Cell and Molecular Biology, Uppsala University, Uppsala, Sweden

[6] These authors contributed equally

* Correspondence: kazum@nips.ac.jp (K.Mu.), chsong@nips.ac.jp (C.S.)

Address: National Institute for Physiological Sciences, 38 Nishigonaka, Myodaiji, Okazaki, Aichi, 444-8585, Japan

Phone: +81-564-55-7893 / FAX: +81-564-55-7895

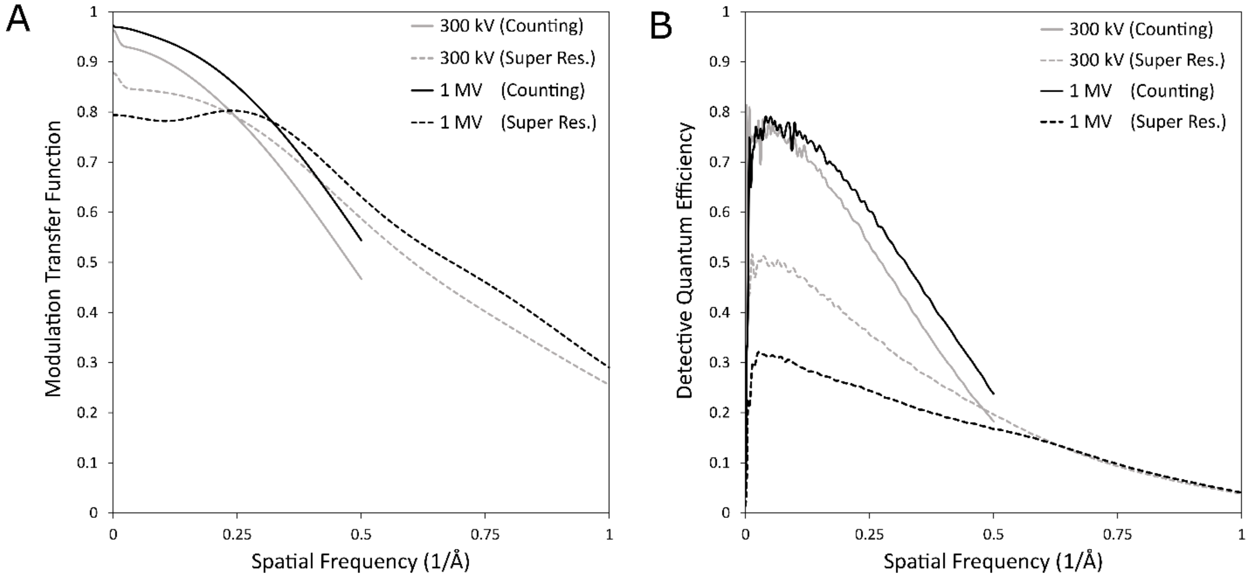

Figure S1. Performance of the detector depending on accelerating voltage. A) Modulation transfer function (MTF) curves for the 1MV cryo-HVEM (JEOL JEM-1000EES) equipped with a K2 Summit direct electron detector (Gatan Inc.) using electron counting and super resolution modes (solid and dashed lines, respectively). Data of a 300 kV microscope are included for comparison, using a Titan Krios G2 (Thermo Fisher Scientific) equipped with a K2 Summit direct electron detector (Gatan Inc.). B) Detective quantum efficiency (DQE) curves for the same conditions as (A).

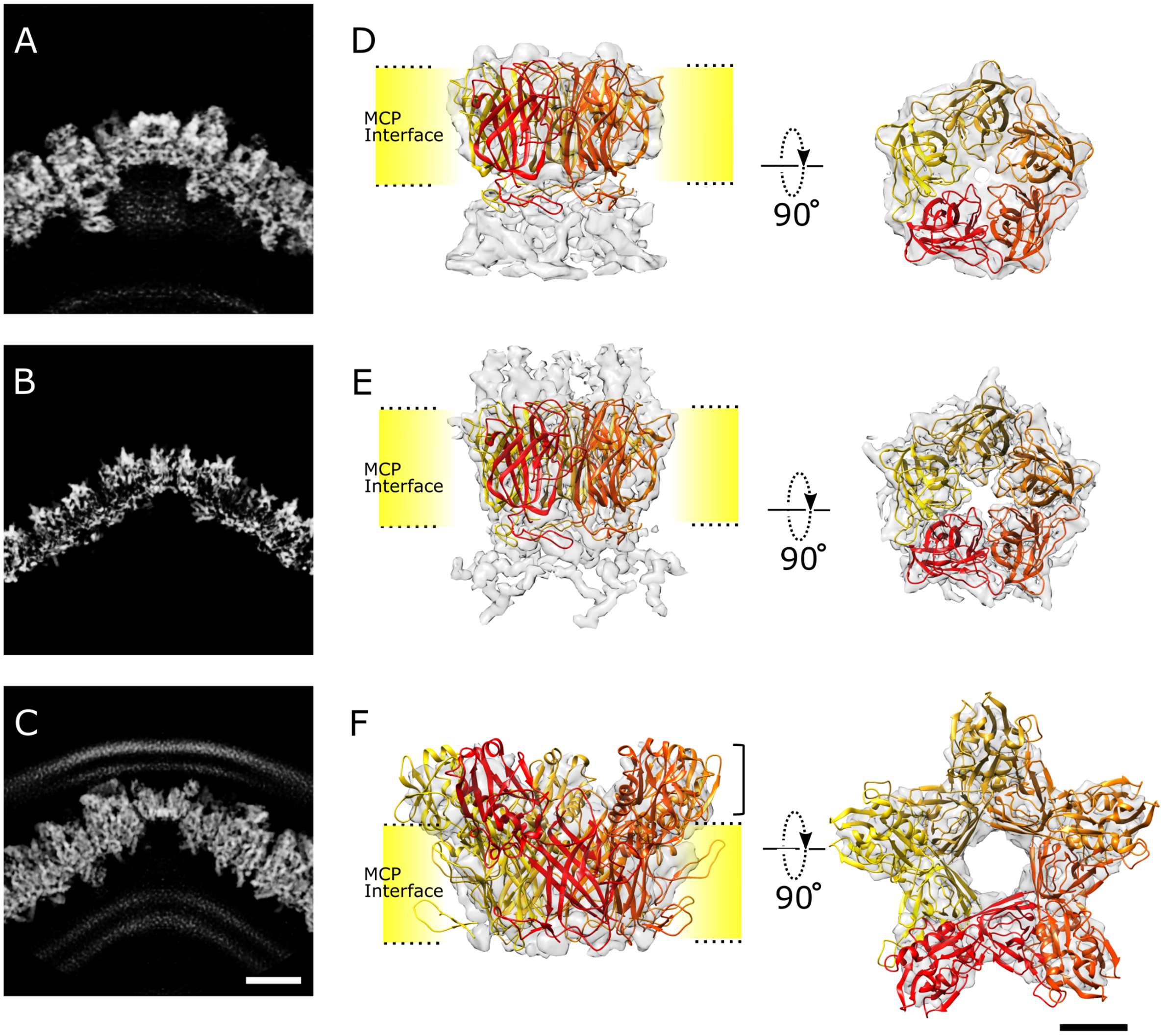

Figure S2. Evaluation of penton structure. Comparing the slab view of tokyovirus pentasymmetron including penton (A) with that of PBCV-1 (B) [1] and ASFV (C) [2]. D) Fitting of the PBCV-1 penton structure (PDBID: 6NCL) to the extracted penton volume of tokyovirus. E) PBCV-1 penton structure (PDBID: 6NCL) fitted to the PBCV-1 penton cryo-EM map (EMD-0436). F) ASFV cryo-EM map (EMD-0815) with the Cafeteriavirus-dependent mavirus penton crystal structure (PDBID: 6G41) [3] fitted and modified. Bracket indicates the insertion domains of the ASFV penton proteins. Scale bars equal 10 nm in slab views and 2 nm in isosurface/model views. The isosurface is shown at 2 σ.

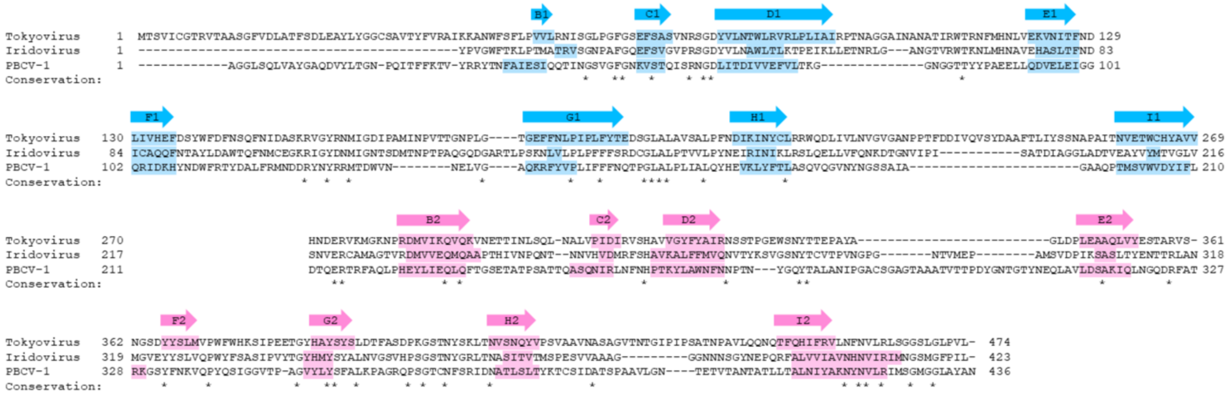

Figure S3. Sequence alignment of Tokyovirus MCP [4] versus iridovirus [5] and PBCV-1 [1]. Putative β-sheets (coloured arrows) were highlighted. Residues conserved between all three sequences are marked with asterisks.

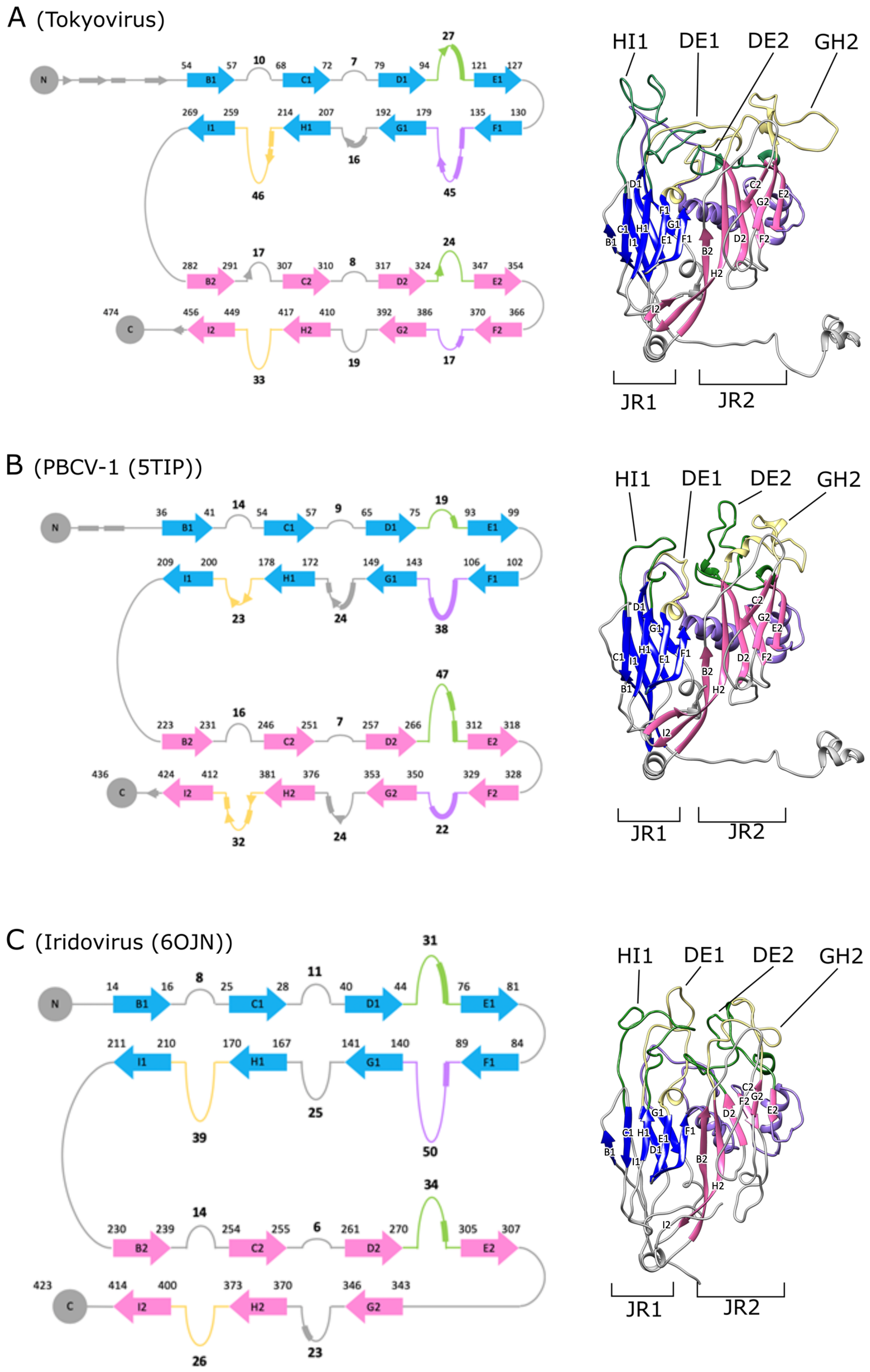
A (Tokyovirus)
HI1
DE1
DE2
GH2
JR1
JR2
B (PBCV-1 (5TIP))
HI1
DE1
DE2
GH2
JR1
JR2
C (Iridovirus (6OJN))
HI1
DE1
DE2
GH2
JR1
JR2

Figure S4. Comparing the secondary structure of MCP monomers. A) Tokyovirus [4], B) PBCV-1 [1], C) Iridovirus [5]. For each, jelly roll motif 1 is coloured in blue, jelly roll motif 2 is coloured in pink, DE1 and DE2 loops are coloured in green, FG1 and FG2 loops are coloured in mauve, HI1 and HI2 loops are coloured in pale yellow, other regions coloured in grey.

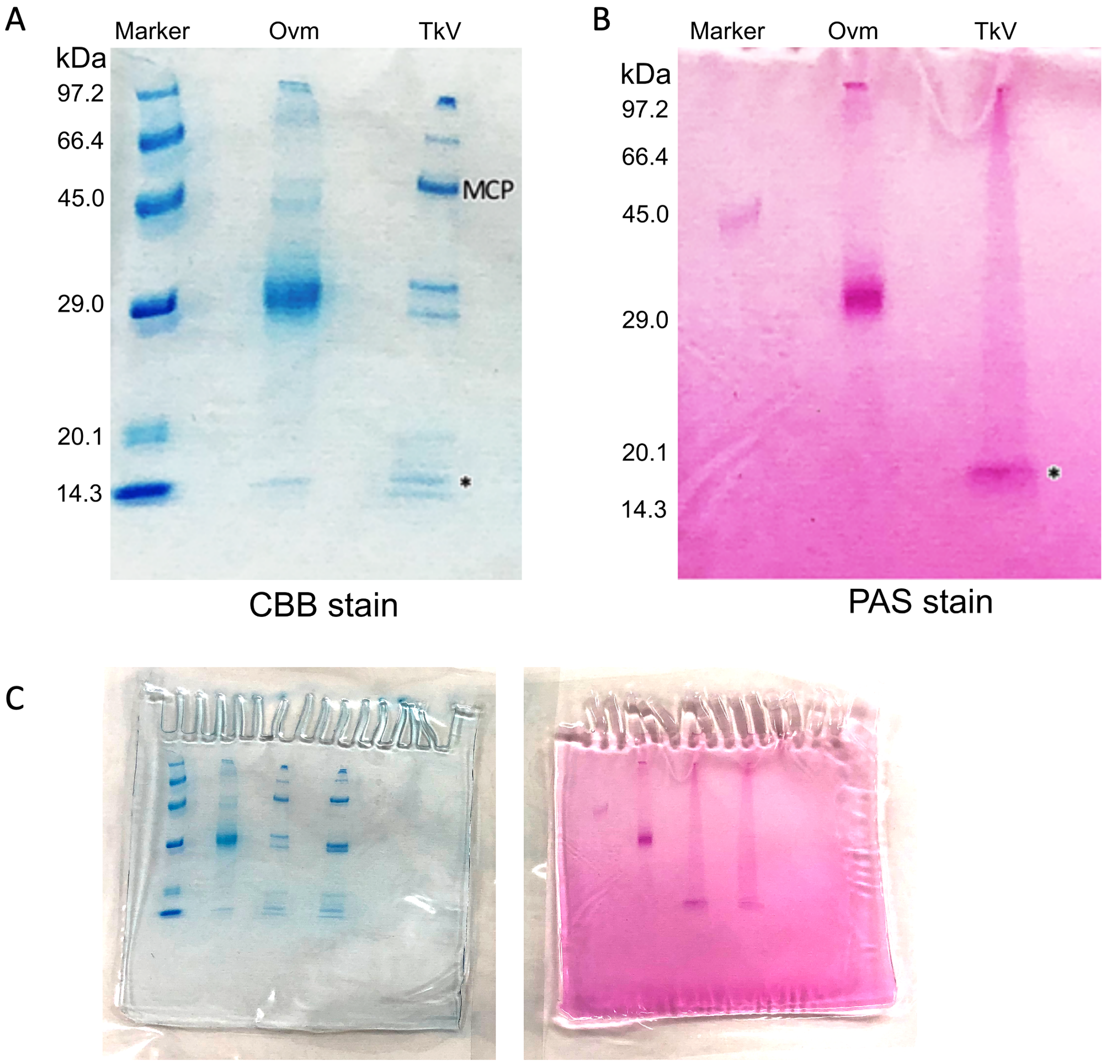

Figure S5. Identification of potential glycoproteins by Periodic acid Schiff (PAS) [6] staining. A) SDS-PAGE gel of a positive control, albumin (Ovm), purified tokyovirus (TkV), with strong bands for TkV MCP indicated at ~52 kDa. The gel was stained with Coomassie Brilliant Blue (CBB). B) PAS stained gel with identical loading to that of (A). In the PAS stained gel, no signal is detected at the same MW as the MCP. A single band is identified by PAS staining for TkV at ~14 kDa (marked with asterisks on both gels). The molecular weights of each marker component is indicated on the left of each gel. C) Original gel images of (A) and (B).

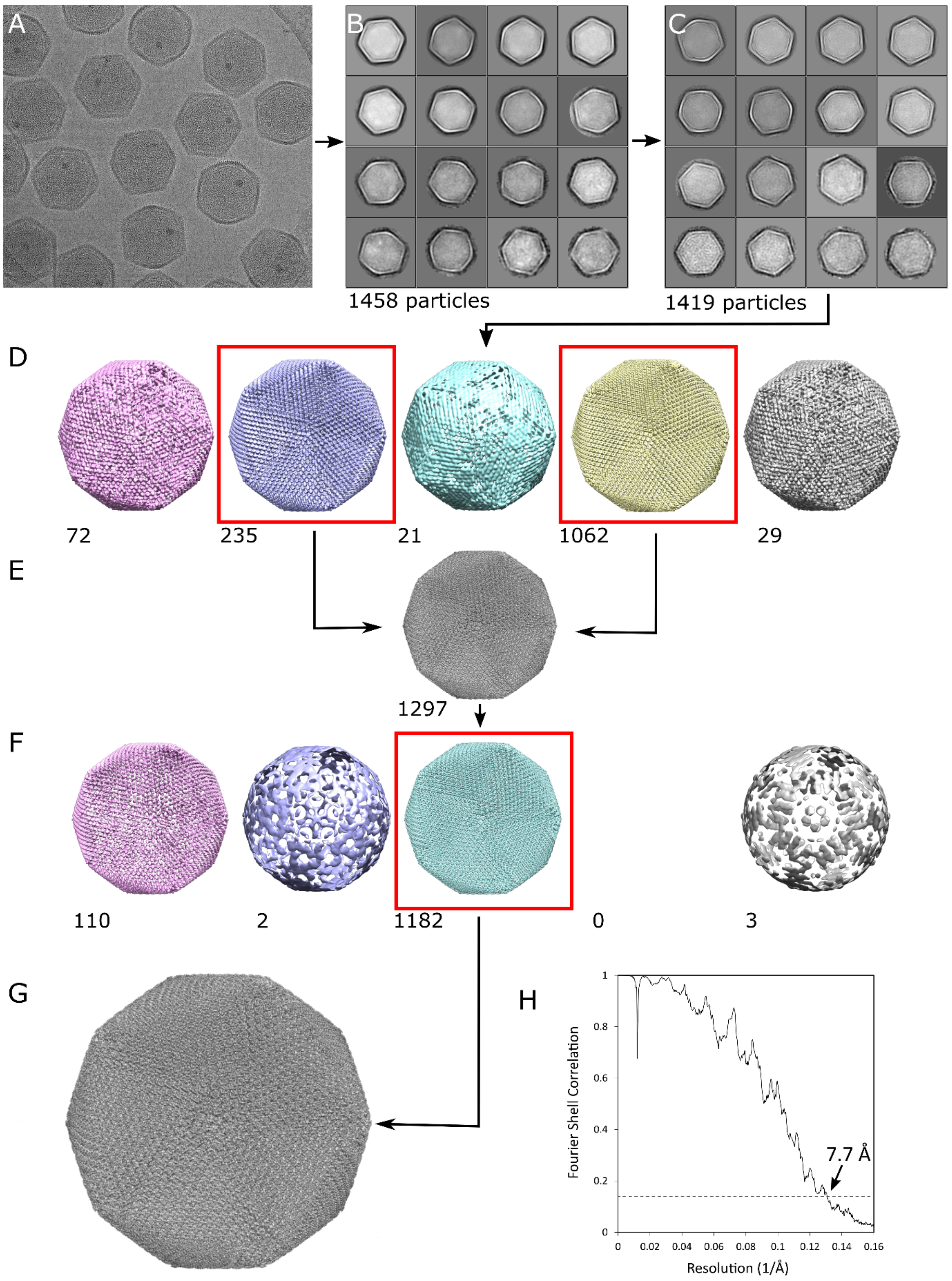
A
B
C
1458 particles
1419 particles
D
72
235
21
1062
29
E
1297
F
110
2
1182
0
3
G
H
Fourier Shell Correlation
Resolution (1/Å)
7.7 Å
0
0.2
0.4
0.6
0.8
1
0.02
0.04
0.06
0.08
0.1
0.12
0.14
0.16

Figure S6. The pathway through 1 MV cryo-HVEM SPA 3D reconstruction of tokyovirus using RELION 3.1 [7]. A) A representative micrograph. The dark spot called “large dense body” peculiar to the Marseilleviruses [8] is clearly observed in individual virus particles. B) First round of 2D classification. C) Second round of 2D classification. D) 3D classification into five classes with the clearest two chosen for initial 3D refinement. E) Initial 3D refinement. F) After CTF refinement cycles, the reconstruction was again classified into five classes, with the highest resolution one chosen for final 3D refinement. G) Final reconstruction. Numbers by a 3D class indicate particle count. A red box indicates class brought forward through processing. H) Gold standard FSC [9] of the final reconstruction.

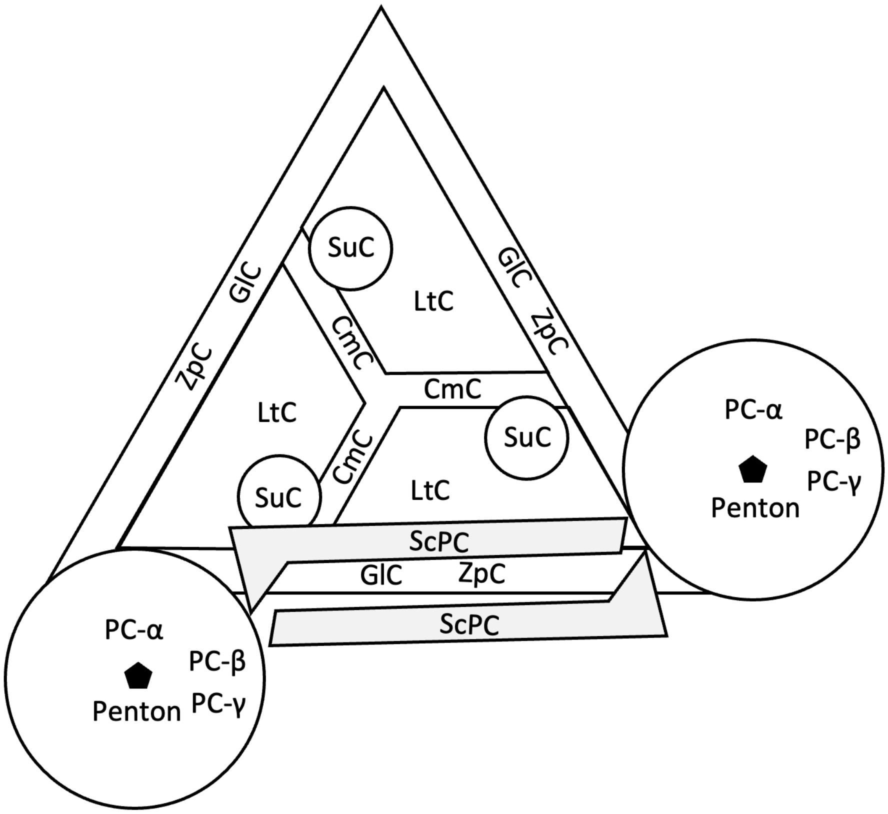

Figure S7. Schematic representation of tokyovirus mCPs, ScPCs, and penton localization, corresponding to Figure 4B. mCPs were classified into 8 components based on the structures, consisted of lattice component (LtC), support component (SuC), cement component (CmC), zipper component (ZpC), glue component (GlC), and 3 pentasymmetron components (PC-α, β, and γ).

SI References

1. Fang, Q. *et al.* Near-atomic structure of a giant virus. *Nat Commun* **10**, 388 (2019).
2. Wang, N. *et al.* Architecture of African swine fever virus and implications for viral assembly. *Science (1979)* **366**, 640–644 (2019).
3. Born, D. *et al.* Capsid protein structure, self-assembly, and processing reveal morphogenesis of the marine virophage mavirus. *Proc Natl Acad Sci U S A* **115**, 7332–7337 (2018).
4. Takemura, M. Infection and Proliferation of Giant Viruses in Amoeba Cells. *Uirusu* **66**, 135–146 (2016).
5. Pintilie, G. *et al.* Segmentation and Comparative Modeling in an 8.6-Å Cryo-EM Map of the Singapore Grouper Iridovirus. *Structure* **27**, 1561-1569.e4 (2019).
6. Aterman, K. & Norkin, S. The periodic acid - Schiff reaction. *Nature* **197**, 1306 (1963).
7. Zivanov, J., Nakane, T. & Scheres, S. H. W. Estimation of high-order aberrations and anisotropic magnification from cryo-EM data sets in RELION-3.1. *IUCrJ* **7**, 253–267 (2020).
8. Okamoto, K. *et al.* Cryo-EM structure of a Marseilleviridae virus particle reveals a large internal microassembly. *Virology* **516**, 239–245 (2018).
9. Scheres, S. H. W. RELION: Implementation of a Bayesian approach to cryo-EM structure determination. *J Struct Biol* **180**, 519–530 (2012).
